# Optical valley separation in two-dimensional semimetals with tilted Dirac cones

**DOI:** 10.1038/s41598-023-45940-4

**Published:** 2023-11-06

**Authors:** Andrew Wild, Eros Mariani, M. E. Portnoi

**Affiliations:** Physics and Astronomy, University of Exeter, Stocker Road, Exeter, EX4 4QL United Kingdom

**Keywords:** Electronic properties and materials, Surfaces, interfaces and thin films

## Abstract

Quasiparticles emerging in crystalline materials can possess a binary flavor known as the valley quantum number which can be used as a basis to encode information in an emerging class of valleytronic devices. Here we show that two-dimensional semimetals with tilted Dirac cones in the electronic band structure exhibit spatial separation of carriers belonging to different valleys under illumination. In stark contrast to gapped Dirac materials this optovalleytronic phenomenon occurs in systems with intact inversion and time-reversal symmetry that host gapless Dirac cones in the band structure, thereby retaining the exceptional graphene-like transport properties. We thus demonstrate that optical valley separation is possible at arbitrarily low photon frequencies including the deep infrared and terahertz regimes with full gate tunability via Pauli blocking. As a specific example of our theory, we predict tunable valley separation in the proposed two-dimensional tilted Dirac cone semimetal 8-*Pmmn* borophene for incident infrared photons at room temperature. This work highlights the potential of two-dimensional tilted Dirac cone materials as a platform for tunable broadband optovalleytronic applications.

## Introduction

Together with familiar degrees of freedom such as charge, momentum and spin, quasiparticles in crystals can possess an additional flavor known as a valley quantum number arising from the underlying lattice symmetry. The most celebrated example are electrons in graphene which behave as massless relativistic quasiparticles described by two inequivalent Dirac cones in the electronic band structure (labeled as $\xi = +$ and − valleys). As the valleys in graphene are widely separated in crystal momentum, carriers rarely scatter between them in the absence of atomic-scale impurities and can thus be sufficiently labeled according to their valley index. There is a rising prospect of using this valley quantum number as a basis to encode information in new valleytronic devices where quasiparticles of a given valley are selected, transported effectively through the material, and detected^1–7^.

The pseudo-relativistic nature of electrons bestows graphene with exceptional transport properties in the form of high electron mobility stemming from the suppression of back-scattering resulting from the Klein tunneling phenomenon^8, 9^. Whilst these transport properties combined with the topological protection of the valley quantum number might suggest graphene to be the ideal platform for valleytronics, practical devices suffer from valley mixing at the sample edges^10, 11^. One way to overcome this limitation is to optically generate valley carriers away from the sample edges. This prompted the exploration of valley control via circularly polarized optical excitations in gapped Dirac cone materials^12, 13^. This optovalleytronic phenomenon is known as the valley Hall effect and was first observed in the direct-gap semiconductor $\text {MoS}_2$^14^. The valley Hall effect upholds the contemporary paradigm of optovalleytronics that optical control over the valley degree-of-freedom is only possible in materials with an electronic band gap. However, these materials: *i*) typically suffer from low electron mobility that hampers the efficiency of information transport across the device and *ii*) due to the presence of the gap it is inherently impossible to obtain optical control at low photon frequencies.

**Figure 1:**
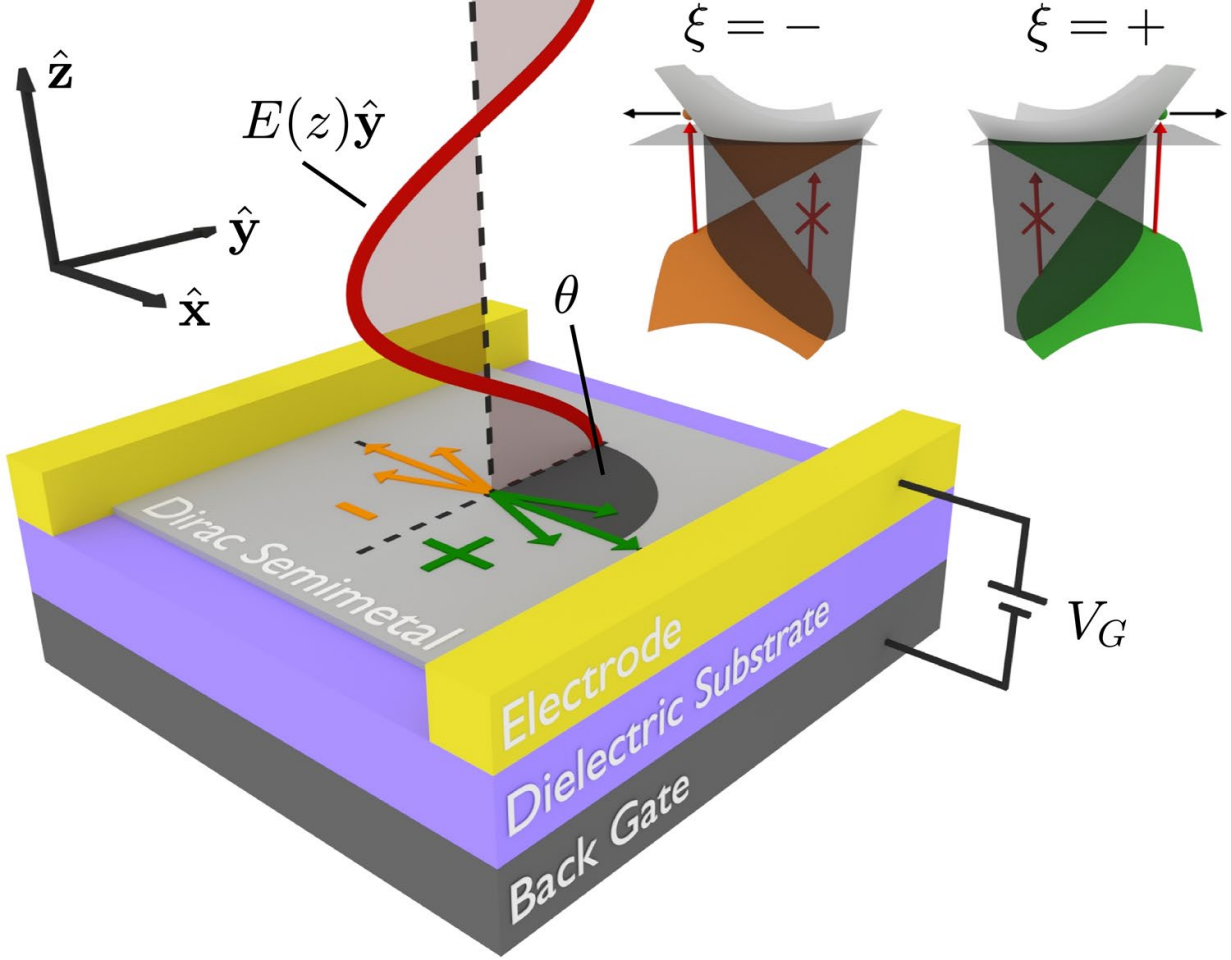
Schematic of the suggested experimental setup for optically generating valley carrier separation in 2D tilted Dirac cone materials. A back-gate configuration with gate voltage $V_\text {G}$ can be used to the change the Fermi level $E_\text {F}$. Linearly polarized photons are described by an electric field which propagates along the $\hat{{\textbf {z}}}$ direction and is polarized at angle $\theta$ to the crystallographic $\hat{{\textbf {x}}}$ axis. The inset shows the band structure of two tilted Dirac cones with valley index $\xi = \pm$ (sketched in green and orange). The incident photons induce interband transitions - in the shaded regions optical transitions are Pauli blocked. The resulting group velocity of photoexcited carriers depends on their valley index.

In this work we provide a solution to these problems: demonstrating the spatial separation of photoexcited carriers at arbitrarily low photon energy belonging to different valleys in two-dimensional (2D) semimetals hosting tilted gapless Dirac cones in the electronic band structure (see Fig. 1). In stark contrast to graphene, the inequivalent valleys in tilted Dirac cone materials are skewed in opposite directions; we exploit this property to optically access the valley quantum number without need of an electronic band gap. The gapless nature of tilted Dirac cone materials, in addition to their superior transport properties, offers a host of advantages over their gapped counterparts. The lack of an electronic band gap ensures that there is no low-frequency threshold in the energy of exciting photons thereby supporting excitation from a broad frequency range of photons down to the highly sought-after terahertz regime^15^. As this mechanism of optical valley control is enabled by asymmetry in the band structure, the exciting light need not be polarized. Furthermore, the degree of valley polarization can be controlled via Pauli blocking which in 2D semimetals is readily tuned with a back gate. The spatial separation of photoexcited carriers belonging to different valleys results in an unequal valley population at opposite sides of the illuminating light spot. It is possible to reverse the sign of the valley polarization at a given side of the light spot by moving the Fermi energy from above to below the Dirac point; this can be practically achieved by tuning a back-gate voltage. We suggest that this effect can be detected by measuring the degree of circular polarization of the edge luminescence in a nearby gapped material^12, 13, 16^, which ideally could be the same material with locally broken inversion symmetry.

Tilted Dirac cones can be classified in to one of three varieties defined by the degree of tilt: sub-critically tilted (type-I) with closed elliptical isoenergy contours, critically tilted (type-III) with open parabolic isoenergy contours and super-critically tilted (type-II) with open hyperbolic isoenergy contours. Two-dimensional materials hosting tilted Dirac cones are an ever growing family with candidate materials including 8-*Pmmn* borophene^17–19^, an organic salt $\alpha$-(BEDT-TTF)$_2$I$_3$^20^ and many more^21–31^. As a case study of our work we demonstrate tunable valley separation in the candidate type-I Dirac cone material 8-*Pmmn* borophene upon illumination of infrared photons at room temperature. In type-II Dirac cone materials, the super-critically tilted band structure results in the perfect spatial separation of valley carriers for a broad frequency range of unpolarized photons for arbitrary Fermi energies. As an extension to our theory we show that type-III Dirac cones will display enhanced spatial separation of valley carriers and emission of highly polarized terahertz photons via hot luminescence aided by the inclusion of carrier scattering.

## Model

We consider a 2D Dirac semimetal with tilted Dirac cones in the electronic band structure described by the Bloch Hamiltonian

1 $$\begin{aligned} H^\xi ({\textbf {q}}) = \hbar v_\text {F} \big ( \xi \gamma \eta q_x \mathbbm {1} + \xi \eta q_x \sigma _x + q_y \sigma _y \big ), \end{aligned}$$

where $\sigma _x$ and $\sigma _y$ are Pauli matrices, $\mathbbm {1}$ is the $2 \times 2$ identity matrix and $v_\text {F}$ is the Fermi velocity along $q_y$ where ${\textbf {q}} = (q_x, q_y)$ is the wavevector measured from the Dirac point in the Brillouin zone corresponding to the inequivalent valleys $\xi = \pm$. The Dirac Hamiltonian has a tilt parameter $\gamma$ which defines sub-critically tilted ($\left| \gamma \right| < 1$, type-I), critically tilted ($\left| \gamma \right| = 1$, type-III) and super-critically tilted ($\left| \gamma \right| > 1$, type-II) Dirac cones. The anisotropy parameter $\eta$ scales the Dirac cone along the tilt axis. Without loss of generality we restrict the Fermi velocity and anisotropy parameter to positive values. The valley-dependent eigenenergies and eigenvectors of the Hamiltonian are defined as

2 $$\begin{aligned} E^\xi _\pm ({\textbf {q}}) = \hbar v_\text {F} q \Big [ \xi \gamma \eta \cos (\varphi _{\textbf {q}}) \pm \sqrt{\eta ^2 \cos ^2(\varphi _{\textbf {q}}) + \sin ^2(\varphi _{\textbf {q}})} \Big ], \end{aligned}$$

and

3 $$\begin{aligned} |\Psi ^\xi _\pm ({\textbf {q}})\rangle = \frac{1}{\sqrt{2}} \begin{bmatrix} \pm \frac{\xi \eta \cos (\varphi _{\textbf {q}}) - i \sin (\varphi _{\textbf {q}})}{\sqrt{\eta ^2 \cos ^2(\varphi _{\textbf {q}}) + \sin ^2(\varphi _{\textbf {q}})}} \\ 1 \end{bmatrix} \end{aligned}$$

respectively, for the conduction ($+$) and valence (−) bands. Here we have defined the wavevector in polar coordinates as $q_x = q\cos (\varphi _{\textbf {q}})$ and $q_y = q \sin (\varphi _{\textbf {q}})$ with *q* the radial wavevector and $\varphi _{\textbf {q}}$ the wavevector angle. The semimetal has a Fermi level $E_\text {F}$ that can be tuned by means of a metallic back gate as shown in Fig. 1. The sample is incident upon by linearly polarized photons with polarization $\hat{{\textbf {e}}}_\theta = \cos (\theta )\hat{{\textbf {x}}} + \sin (\theta )\hat{{\textbf {y}}}$ and energy $h \nu$. We treat the corresponding electric field as a time-dependent perturbation to the otherwise time-independent system using Fermi’s golden rule inducing vertical, interband transitions. In this work we do not consider intraband absorption as it requires knowledge of material-dependent scattering mechanisms and in the case of type-II Dirac cone materials, a detailed understanding of the Fermi surface beyond the Dirac cone approximation. We also note that our mechanism works for photons incident normally on the sample and does not rely on in-plane momentum transfer to electrons via phenomena such as photon-drag^32^.

**Figure 2:**
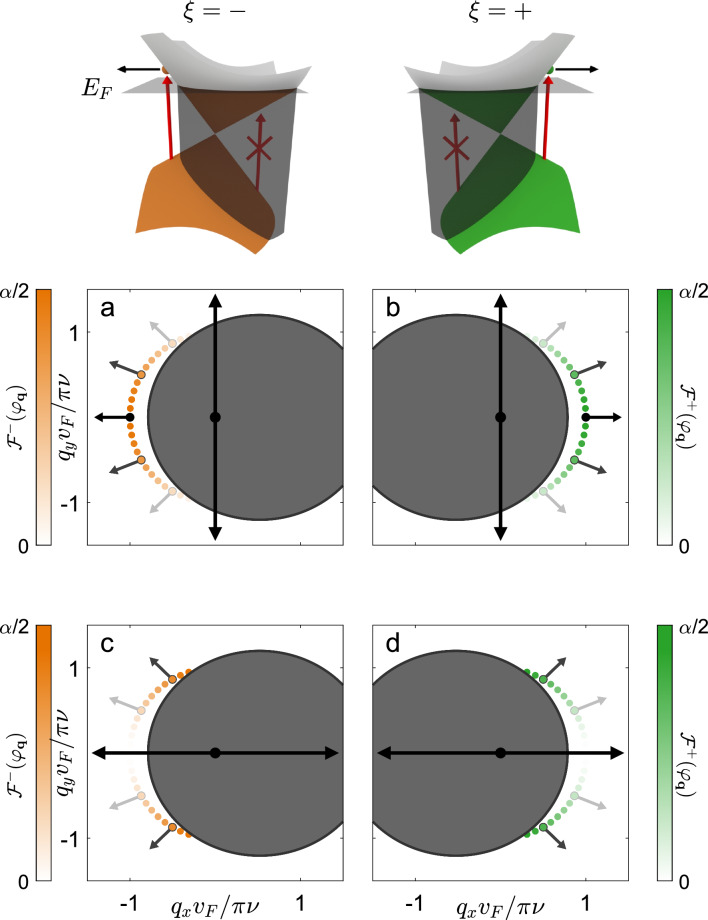
Distribution of photoexcited electrons in a type-I Dirac cone material characterized by tilt parameter $\gamma = 0.4$ and anisotropy parameter $\eta = 1$. The incident light consists of photons with energy $h\nu$ polarized along the crystallographic $\hat{{\textbf {y}}}$ (panels a and b) and $\hat{{\textbf {x}}}$ (panels c and d) axes. The Fermi energy is fixed relative to the photon energy $E_\text {F} = 0.55h\nu$. Photoexcited electrons fall on the perimeter of a circle in wavevector space with radius $\pi \nu /v_\text {F}$. The distribution of photoexcited electrons $\mathcal {F}^\xi (\varphi _{\textbf {q}})$ depends on the wavevector angle $\varphi _{\textbf {q}}$ and is sketched as a dotted line with color coded magnitude: green for valley $\xi = +$ and orange for valley $\xi = -$. The regions of Pauli blocked optical transitions are superimposed as gray ellipses. The black arrows reflect the direction and magnitude of the group velocity of photoexcited electrons ${\textbf {v}}^\xi _g(\varphi _{\textbf {q}})$ for a selection of wavevector angles.

There are three factors that govern the optical absorption of photons. *i*) Initial and final states with wavevector ${\textbf {q}}$ must be separated by an energy of $\Delta E({\textbf {q}}) = E^\xi _+({\textbf {q}}) - E^\xi _-({\textbf {q}}) = h \nu$. For a fixed frequency $\nu$ this condition gives a set of wavevectors available for the transition given by

4 $$\begin{aligned} \Delta E({\textbf {q}}) = 2 \hbar v_\text {F} q \sqrt{\eta ^2 \cos ^2(\varphi _{\textbf {q}}) + \sin ^2(\varphi _{\textbf {q}})}. \end{aligned}$$

It can be seen that the states contributing to absorption fall on the perimeter of an ellipse in wavevector space with semi-major and semi-minor axes ($\pi \nu / v_\text {F}$ and $\pi \nu / \eta v_\text {F}$) proportional to the frequency of the incident photon. For the case of the anisotropy parameter equaling unity ($\eta = 1$), this ellipse becomes a circle with radius $\pi \nu /v_\text {F}$. The geometry of this ellipse is independent of both the valley index ($\xi$) and tilt parameter ($\gamma$). *ii*) The transition rate describes the likelihood of an absorption event occurring at a given wavevector. For linearly polarized photons the transition rate is proportional to the absolute value squared of the expectation value of the velocity operator projected along the axis of polarization ($v_{\text {cv}}({\textbf {q}}) = \langle \Psi ^\xi _\pm ({\textbf {q}})| \hat{{\textbf {e}}}_\theta \cdot {\textbf {v}} |\Psi ^\xi _\mp ({\textbf {q}})\rangle$) between the initial and final states^33–35^. Here, we utilize the velocity operator defined within the gradient approximation ${\textbf {v}} = (1/\hbar ) \varvec{\nabla }_{\textbf {q}} H^\xi ({\textbf {q}})$ which is valid in the vicinity of a band degeneracy, i.e., Dirac point and when the initial and final states originate from the same atomic orbitals (see, e.g., Ref.^36^). We can now derive the squared velocity matrix element yielding

5 $$\begin{aligned} \mid v_{\text {cv}}(\varphi _{\textbf {q}}) \! \mid ^2 \, = \frac{\eta ^2 v_\text {F}^2}{\eta ^2 \cos ^2(\varphi _{\textbf {q}}) + \sin ^2(\varphi _{\textbf {q}})}\sin ^2(\varphi _{\textbf {q}} - \theta ). \end{aligned}$$

The formula above demonstrates the optical momentum alignment phenomenon in Dirac materials in which, upon absorption of photons polarized along $\theta$, carriers are generated with wavevector angle $\varphi _{\textbf {q}}$ predominantly perpendicular to the polarization vector^34, 35, 37^. The velocity matrix element is equivalent to that of non-tilted cones and hence is independent of both valley ($\xi$) and tilt parameter ($\gamma$). *iii*) For an absorption event to occur we must ensure that the initial state is occupied and the final state is empty to avoid Pauli blocking. We define these conditions with the Fermi-Dirac distributions $f_e(E) = 1 - f_h(E) = \big \{ 1 + \exp \big [ (E - \mu )/k_\text {B} T \big ] \big \}^{-1}$ for electrons (*e*) and holes (*h*) with Boltzmann constant $k_\text {B}$, chemical potential $\mu$ and temperature *T*. Crucially, the regions of Pauli blocking are valley ($\xi$) and tilt parameter ($\gamma$) dependent leading to valley-dependent distributions of photoexcited carriers for certain values of the Fermi energy.

Combining all of these factors we can write the angular distribution of excited carriers^34, 35^ at the instant of photocreation as

6 $$\begin{aligned} \mathcal {F}^\xi (\varphi _{\textbf {q}}) = \alpha \frac{g_\text {s} \hbar }{2 \pi \nu } \mid \! v_\text {cv}(\varphi _{\textbf {q}}) \! \mid ^2 \int _{0}^\infty \!\!\!\!\!\! \delta \big [ \Delta E({\textbf {q}}) - h \nu \big ] f_e \big [E^\xi _-({\textbf {q}})\big ] f_h \big [E^\xi _+({\textbf {q}})\big ] q \text {d} q, \end{aligned}$$

where $g_\text {s} = 2$ accounts for the spin degeneracy, $\delta [...]$ is the Dirac delta function and $\alpha = e^2/\hbar c \approx 1/137$ is the fine-structure constant in CGS units. The Dirac delta function in Eq. (6) ensures that the photoexcited electrons fall on the perimeter of an ellipse in wavevector space with geometry dictated by Eq. (4). Therefore, the angular distribution of photoexcited carriers is defined in terms of the wavevector angle $\varphi _{\textbf {q}}$ of photoexcited electrons falling on this ellipse.

**Figure 3:**
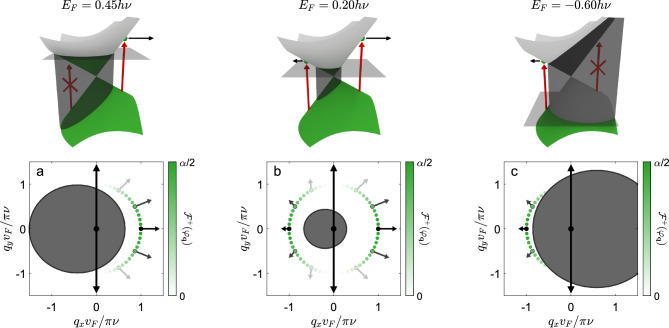
Distribution of photoexcited electrons in a type-I Dirac cone material characterized by tilt parameter $\gamma = 0.4$ and anisotropy parameter $\eta = 1$. The incident light consists of photons with energy $h\nu$ polarized along the crystallographic $\hat{{\textbf {y}}}$ axis. In this figure, the Fermi energy takes the values: $E_\text {F} = 0.45h\nu$ in panel a, $E_\text {F} = 0.20h\nu$ in panel b, and $E_\text {F} = -0.60h\nu$ in panel c. Photoexcited electrons fall on the perimeter of a circle in wavevector space with radius $\pi \nu /v_\text {F}$. The distribution of photoexcited electrons $\mathcal {F}^\xi (\varphi _{\textbf {q}})$ depends on the wavevector angle $\varphi _{\textbf {q}}$ and is sketched as a dotted line with color coded magnitude. Here we only plot the distribution of photoexcited carriers for a single valley $\xi = +$. In each case the regions of Pauli blocked transitions are superimposed as gray ellipses. The black arrows reflect the direction and magnitude of the group velocity of photoexcited electrons ${\textbf {v}}^\xi _g(\varphi _{\textbf {q}})$ for a selection of wavevector angles.

We note that the normalization factor is chosen such that integrating over the wavevector angle yields the ratio of absorbed photons. Accordingly, the absorption is defined as $\mathcal {A} = g_\text {v} \int _0^{2\pi } \mathcal {F}^\xi (\varphi _{\textbf {q}}) \text {d} \varphi _{\textbf {q}}$, where $g_\text {v} = 2$ is the valley degeneracy^15^. Note that for the case of graphene ($\gamma = 0$ and $\eta = 1$) this expression simplifies to the well-known universal sheet absorption of $\mathcal {A} = \pi \alpha \approx 2.3\%$. For the full analytic expression of the distribution of photoexcited carriers $\mathcal {F}^\xi (\varphi _{\textbf {q}})$ see the supplementary material. We note that the absorption of tilted Dirac cone materials can equally obtained using the Kubo formalism^38^, the agreement of these two prescriptions confirms the correctness of each approach.

## Results

### Type-I Dirac cones

Initially, we consider a type-I ($\left| \gamma \right| < 1$) Dirac cone material with Fermi level sitting above the Dirac point (see Fig. 2). For incident photons polarized along the crystallographic $\hat{{\textbf {y}}}$ axis ($\theta = \pi /2$), the optical momentum alignment phenomenon dictates that photoexcited electrons are created close to the tilt ($q_x$) axis of the Dirac cone. However, any state inside the Pauli blocked regions does not undergo a transition. The resulting distribution of photoexcited electrons shows that if the tilt parameter takes on a positive value ($\gamma > 0$) the majority of electrons in valley $\xi = +$ (−) are created on the right (left) side of the Dirac cone (see Fig. 2a,b). The group velocities resulting from the conical band structure dictate that at photocreation, electrons with valley number $\xi = +$ will propagate to the right ($\hat{{\textbf {x}}}$ direction) whilst electrons with valley number $\xi = -$ will propagate to the left ($-\hat{{\textbf {x}}}$ direction) towards the different sides of the illuminated light spot. We note that in general, the tilt parameter could take a negative value ($\gamma < 0$), in this case electrons from valley $\xi$ will propagate in the $-\xi \hat{{\textbf {x}}}$ direction.

Although we have highlighted spatial separation of photoexcited electrons from different valleys for a specific polarization ($\theta = \pi /2$), this phenomenon occurs, to a lesser extent, for all polarizations. As the polarization plane is rotated towards the crystallographic $\hat{{\textbf {x}}}$ axis ($\theta = 0$) an increased amount of electrons move along the $\hat{{\textbf {y}}}$ axis, nevertheless, there is still a significant amount of valley separation (see Fig. 2c,d). As it is possible to determine the orientation of the crystallographic axes of a Dirac semimetal with an optical procedure^15^, aligning the incident photon polarization close to $\hat{{\textbf {y}}}$ will yield optimal results.

By modifying the Fermi level we can change the distribution of photoexcited electrons and control the degree of valley polarization at the sides of the light spot. For example, by placing the Fermi level above the Dirac point we can ensure that electrons from valley $\xi = +$ propagate to the right side of the light spot ($\hat{{\textbf {x}}}$ direction) whilst electrons from valley $\xi = -$ propagate to the left side of the light spot ($-\hat{{\textbf {x}}}$ direction) as demonstrated in Fig. 3a. If the Fermi level sits near to the Dirac point then no Pauli blocking occurs and electrons from both valleys propagate to either side of the light spot as seen in Fig. 3b. Moving the Fermi level below the Dirac point flips the propagation direction of photoexcited carriers with electrons from valley $\xi$ moving in the $-\xi {\textbf {x}}$ direction towards opposite sides of the light spot as seen in Fig. 3c.

To quantify the valley separation we define the parameter $\mathcal {N}_{R(L)}^\xi$ to be the percentage of photoexcited electrons in valley $\xi$ that propagate to the right (left) side of the light spot along the crystallographic $\hat{{\textbf {x}}}$ axis

7 $$\begin{aligned} \mathcal {N}^\xi _R = \frac{\int _\Sigma \mathcal {F}^\xi (\varphi _{\textbf {q}})\text {d} \varphi _{\textbf {q}}}{\int \mathcal {F}^\xi (\varphi _{\textbf {q}})\text {d} \varphi _{\textbf {q}}}. \end{aligned}$$

The domain of integration $\Sigma$ is defined as the set of angles $\varphi _{\textbf {q}}$ corresponding to a positive $\hat{{\textbf {x}}}$ component of the group velocity ${\textbf {v}}^\xi _{g}(\varphi _{\textbf {q}}) \cdot \hat{{\textbf {x}}}$ where ${\textbf {v}}^\xi _{g}(\varphi _{\textbf {q}}) = (1/\hbar )\varvec{\nabla }_{\textbf {q}}E^\xi _+({\textbf {q}})$ and $\mathcal {F}^\xi (\varphi _{\textbf {q}})$ is defined by Eq. (6). The parameter $\mathcal {N}^\xi _L$ can be deduced from the normalization condition $\mathcal {N}_R^\xi + \mathcal {N}_L^\xi = 1$. Using these quantities, we can define the degree of valley polarization at the right-hand side of the light spot as

8 $$\begin{aligned} \mathcal {S}_R = \frac{\mathcal {N}^+_{R} - \mathcal {N}^-_{R}}{\mathcal {N}^+_{R} + \mathcal {N}^-_{R}}. \end{aligned}$$

If all photoexcited electrons at the right-hand side of the light spot are from valley $\xi$ then the valley polarization takes on the value $\mathcal {S}_R = \xi$, in contrast, if there is an equal number of electrons from either valley then $\mathcal {S}_R = 0$. The valley polarization at the left-hand side of the light spot is the opposite of the right-hand side $\mathcal {S}_L = -\mathcal {S}_R$. The degree of valley polarization can be detected when photoexcited electrons propagate into a nearby gapped material, where they can recombine emitting circularly polarized photons with handedness determined by their valley index $\xi$. The degree of valley polarization maps on to the degree of circular polarization of the emitted light.

**Figure 4:**
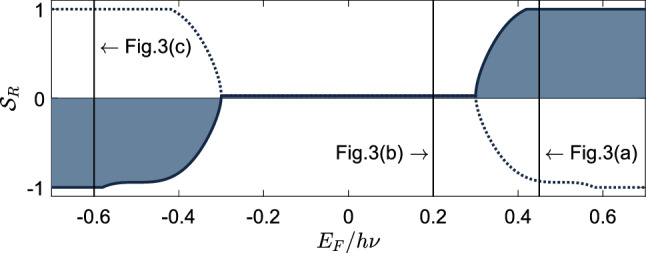
Degree of valley polarization for photoexcited electrons ($\mathcal {S}_R$) is plotted as a function of Fermi energy $E_\text {F}$ normalized by photon energy $h\nu$ (sketched with solid line and shaded). In this figure, the Dirac cone tilt parameter is set to $\gamma = 0.4$, the anisotropy parameter is set to unity $\eta = 1$, and the incident light is polarized along the crystallographic $\hat{{\textbf {y}}}$ axis. The three solid lines at $E_\text {F}/h\nu = 0.45$, 0.20, and $-0.60$ correspond to the parameters used in plots in Fig. 3a,b and c respectively. The degree of valley polarization calculated for holes is sketched in the dotted line.

In Fig. 3 we visually demonstrated that varying the Fermi energy tuned the degree of valley polarization. In Fig. 4 we quantify this effect by plotting the degree of valley polarization $\mathcal {S}_R$ as a function of Fermi energy. Utilizing the analytic expressions for $\mathcal {S}_{R/L}$ for the case of $\theta = \pi /2$ provided in the supplementary material, we can deduce what values of Fermi energy yield a maximal degree of valley polarization, i.e., $\left| S_{R/L} \right| = 1$. When the Fermi level sits above the Dirac point the degree of valley polarization takes the value $\mathcal {S}_R = \text {sign}(\gamma )$ when $h\nu (1 - \left| \gamma \right| ^2)/2< E_\text {F} < h\nu (1 + \left| \gamma \right| )/2$. On the contrary, when the Fermi level sits below the Dirac point the degree of valley polarization takes the value $\mathcal {S}_R = -\text {sign}(\gamma )$ when $-h\nu (1 + \left| \gamma \right| )/2< E_\text {F} < -h\nu (1 + \left| \gamma \right| ^2)/2$. We note that the group velocities resulting from the tilted Dirac cones culminate in an overall preference for carriers in valley $\xi$ to propagate in the $\text {sign}(\xi \gamma )\hat{{\textbf {x}}}$ direction, thereby justifying the asymmetry in Fig. 4 between positive and negative values of $\mathcal {S}_R$. It also noted that whilst the solid line in Fig. 4 plots the degree of valley polarization for photoexcited electrons in the conduction band, the same quantity can be defined in for holes in the valence band. The degree of valley polarization for holes can be obtained from $\mathcal {S}_{R/L}$ by inverting the sign of the Fermi energy ($E_\text {F} \rightarrow -E_\text {F}$) as indicated by the dotted line in Fig. 4. For Fermi energies exceeding the bounds of Fig. 4, i.e., $\left| E_\text {F} \right| > h\nu (1 + \left| \gamma \right| )/2$ there is no absorption^15^ and thus the degree of valley polarization is not defined.

### Special case: 8-*Pmmn* borophene

As a specific case study of our theory we demonstrate the spatial separation of photoexcited electrons from different valleys in the predicted tilted type-I Dirac cone material 8-*Pmmn* borophene. We consider the case of infrared photons with wavelength $\lambda = 10\mu \text {m}$ that are polarized along the crystallographic $\hat{{\textbf {y}}}$ axis. In this material the Dirac cones have a Hamiltonian of the form given in Eq. (1) with Fermi velocity $v_\text {F} = 8.6 \times 10^5\text {ms}^{-1}$, tilt parameter $\gamma = 0.46$ and anisotropy parameter $\eta = 0.80$^19^. Guided by our analytic formulas for $\mathcal {S}_{R/L}$, we find that the degree of valley polarization is maximal $\mathcal {S}_{R} = 1$ when the Fermi energy is set between approximately $50\text {meV}< E_\text {F} < 90\text {meV}$ or $\mathcal {S}_R = -1$ when $-90\text {meV}< E_\text {F} < -75\text {meV}$.

We can also evaluate the degree of valley polarization for the system when at room temperature ($T = 300\text {K}$). In this case, the step-like regions of Pauli blocked transitions are replaced by smooth Fermi-Dirac distributions defined with the chemical potential $\mu$. Assuming that the monolayer is in a back-gate configuration we can tune the degree of valley polarization by varying carrier density from charge neutrality ($\Delta n = \pm 10^{12}\text {cm}^{-2}$). Using the Fermi-Dirac distribution and the density of states of a tilted Dirac cone we can determine that these electronic densities correspond to a chemical potential of $\mu \approx \pm 60$meV for a system at room temperature. Varying the chemical potential between these two values we can modify the degree of valley polarization from $\mathcal {S}_R \approx 0.45$ ($\Delta n = 10^{12}\text {cm}^{-2}$) to $\mathcal {S}_R \approx -0.36$ ($\Delta n = -10^{12}\text {cm}^{-2}$). If the experimental setup allows for larger values of carrier density it will be possible to obtain values for $\mathcal {S}_R$ that are closer to unity at room temperature. Therefore, in one of the most well-known predicted tilted Dirac cone materials, 8-*Pmmn* borophene, we have demonstrated tunable, optical control over the valley degree-of-freedom under illumination from infrared light with room temperature operation.

After initial interband excitation, it is possible to consider energy and momentum relaxation via carrier-phonon scattering. For low-frequency excitation, we can neglect phonon-mediated intervalley scattering as well as intravalley scattering by optical phonons. Within a single valley, carriers will undergo momentum relaxation due to scattering with acoustic phonons, however, quantifying these effects would require knowledge of material-specific scattering rates. Any intermediate state following momentum relaxation is subject to the same Pauli blockade as the initial excitation and will thus not qualitatively impact the spatial separation of valley carriers.

**Figure 5:**
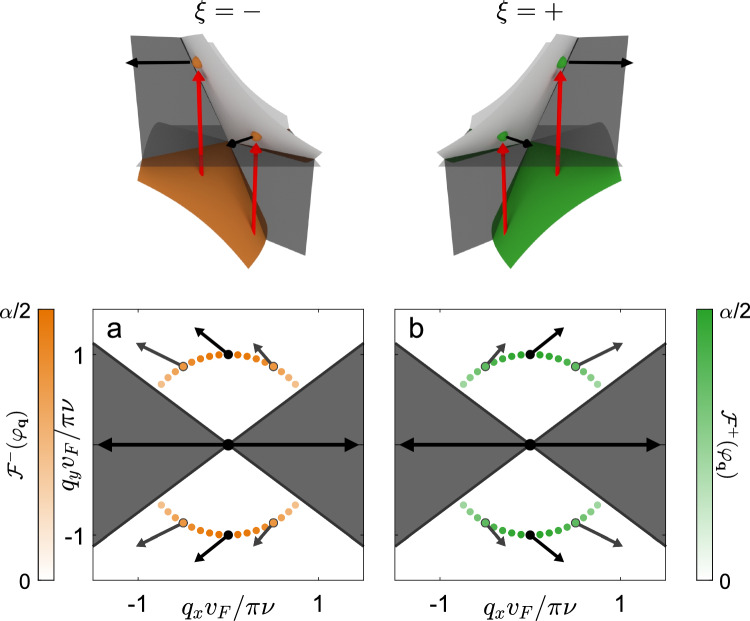
Distribution of photoexcited electrons in a type-II Dirac cone material characterized by tilt parameter $\gamma = 1.25$, anisotropy parameter $\eta = 1$ and Fermi energy $E_\text {F} = 0$. The incident light consists of photons with energy $h\nu$ polarized along the crystallographic $\hat{{\textbf {x}}}$ axis. Photoexcited electrons fall on the perimeter of a circle in wavevector space with radius $\pi \nu /v_\text {F}$. The distribution of photoexcited electrons $\mathcal {F}^\xi (\varphi _{\textbf {q}})$ depends on the wavevector angle $\varphi _{\textbf {q}}$ which is sketched as a dotted line with color coded magnitude: green for valley $\xi = +$ and orange for valley $\xi = -$. The regions of Pauli blocked optical transitions are superimposed in gray. The black arrows reflect the direction and magnitude of the group velocity of photoexcited electrons ${\textbf {v}}^\xi _g(\varphi _{\textbf {q}})$ for a selection of wavevector angles.

### Type-II Dirac cones

Unlike their type-I counterparts, type-II ($\left| \gamma \right| > 1$) Dirac cones are super-critically tilted. The group velocities resulting from the super-critically tilted band structure dictates that all photoexcited electrons will be spatially separated according to their valley index (see Fig. 5). In other words, as long as there is absorption (which requires $E_\text {F} < h \nu (1 + \left| \gamma \right| )/2$) there will always be full spatial separation of photoexcited electrons according to their valley index $\mathcal {S}_{R} = \text {sign}(\gamma )$. As in type-II Dirac cone materials the degree of valley polarization is always maximal, for demonstrative purposes, we pick the polarization of light that maximizes the absorption, i.e., $\theta = 0$.

### Carrier relaxation enhanced momentum alignment in type-III Dirac cones

Up until this point, critically tilted type-III Dirac cones have merely marked the boundary between type-I and II Dirac cones. However, when including the effects of carrier relaxation, type-III Dirac cones offer an interesting mechanism of momentum alignment not possible in any other tilted Dirac cones.

**Figure 6:**
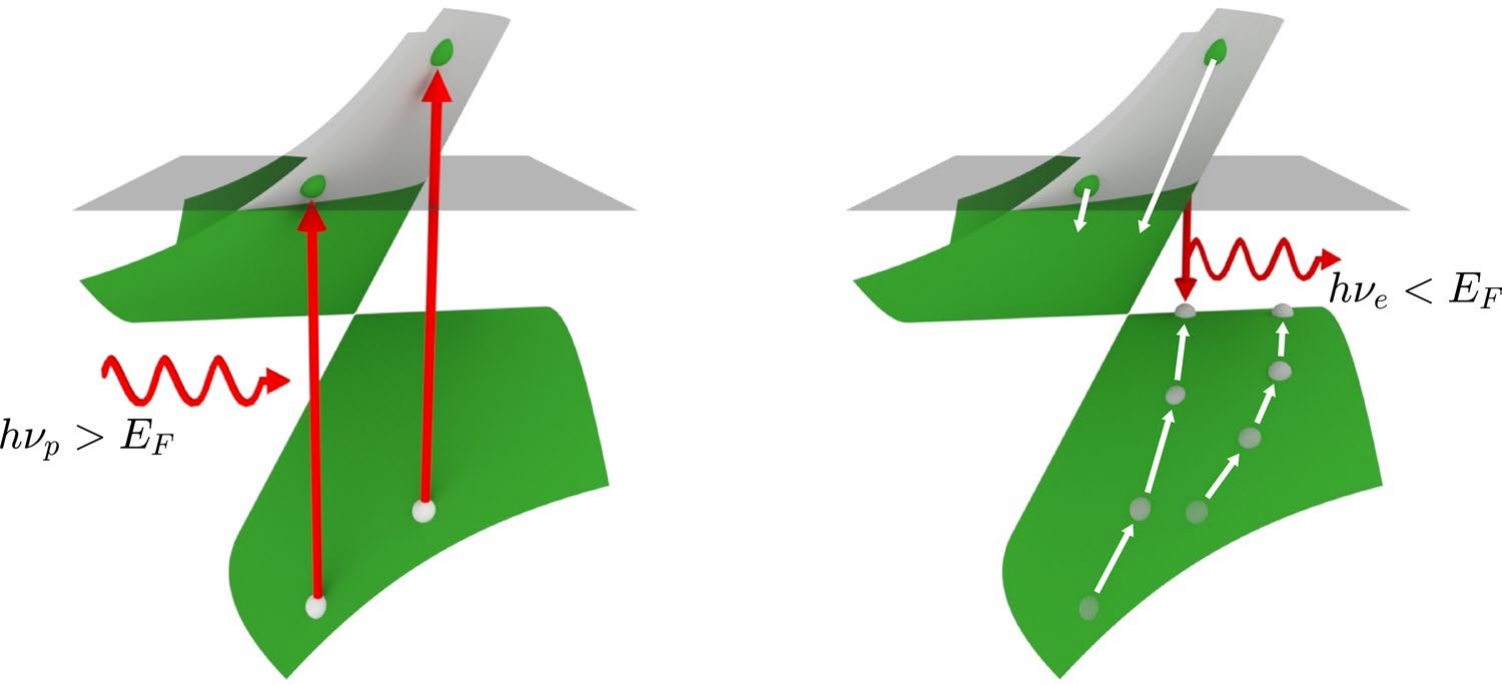
Schematic of enhanced momentum alignment in a type-III Dirac cone with valley index $\xi = +$ and tilt parameter $\gamma = 1$. Red arrows indicate interband absorption/emission and white arrows indicate relaxation via carrier-carrier and carrier-phonon scattering processes. After interband absorption $h \nu > E_\text {F}$ holes float towards the Fermi level becoming trapped in an intermediate state with wavevector $q_y = 0$. Upon recombination photons will be emitted with polarization aligned with the crystallographic $\hat{{\textbf {y}}}$ axis and energy $h \nu < E_\text {F}$.

Critically tilted type-III ($\left| \gamma \right| = 1$) Dirac cones have a peculiar band structure in which the extrema of the upper and lower bands are one-dimensional lines in the wavevector space. First, we pump the material with arbitrarily polarized photons with energy $h \nu _p > E_\text {F}$ (see Fig. 6). The resulting electrons and holes relax via a combination of carrier-carrier and carrier-phonon scattering processes to the most energetically favorable state. The holes aim to increase their energy, floating to the one-dimensional band maxima. The holes become stranded in these intermediate states which are perfectly aligned in momenta. Any holes that relaxed to a small wavevector $\mid q_x \mid$ will be able to recombine with electrons in the upper band emitting photons of energy $h \nu _e < E_\text {F}$. Due to the momentum alignment of these holes, the emitted photons will have polarization aligned with the crystallographic $\hat{{\textbf {y}}}$ axis. This mechanism of emission via an intermediate state is known as hot luminescence^39^. By modifying the Fermi level with a back-gate voltage, the emission energy of these photons can be tuned to the terahertz regime yielding a highly-polarized tunable terahertz emitter.

## Conclusion

The realization of the valley-polarized currents via the valley Hall effect provided the elementary building block for valleytronic devices in gapped Dirac cone materials (see review articles^1–6^ and references therein). This discovery sparked a desire for valleytronic components that in conjunction with the valley Hall effect could lead to valley-sensitive logic gates for classical and quantum computing applications^7^. In our work we demonstrate the spatial separation of valley carriers away from the light spot in gapless Dirac materials with tilted Dirac cones. Our discovery paves the way to the realization of novel valleytronic devices benefiting from the superior transport properties of massless Dirac fermions.

With the recent burst of interest in massless tilted Dirac cone materials there have been several theoretical works investigating the valley-dependent transport of carriers traversing gated junctions, waveguides and external fields^40–44^. Combining these transport techniques with the optical spatial separation of valley carriers proposed in our work could enable the design of valleytronic components such as valley filters and switches in gapless materials. It may also be possible to further direct the propagation of valley carriers across graphene-based interconnects based on electrostatic waveguides^45, 46^, quantum wire leads^47^ or gated junctions in externally applied fields^48^. Furthermore, the spatial separation of valley carriers in gapless tilted Dirac cone materials could be combined with valley-sensitive components of gapped Dirac cone materials such as valley transistors^49^ or decoding the valley index via emission of circularly polarized light^12, 13, 16^. This would require a detailed understanding of the transport phenomena occurring at the interface between gapless and gapped Dirac cone materials. It is well-known that placing graphene on a hexagonal boron nitride substrate induces a superlattice structure inducing local regions with pseudo-gaps^50–52^—a similar technique for tilted Dirac materials should enable the seamless transport of valley carriers between gapless and gapped regions in the spectrum allowing valley index measurement. Whilst we suggest the use of a gapped region of the material to decode the valley index, before measurement, information transfer across the device will benefit from the superior transport properties of massless Dirac fermions as well as gate-tunable optical valley control. Lastly, the theoretical and computational predictions of two-dimensional materials hosting massless tilted Dirac cones are rapidly growing in number^17–31^. The experimental efforts aiming at realizing these materials are catching up^53–55^. We hope that the prospect of optovalleytronics put forward in our work will stimulate further research into massless tilted Dirac cone materials.

### Supplementary Information

Supplementary Information.

## Data Availability

All data generated or analysed during this study are included in this published article and its supplementary information files.
