# Supplementary material for: Optical valley separation in two-dimensional semimetals with tilted Dirac cones
Source: Sci Rep. 2023 Nov 6;13:19211. doi: 10.1038/s41598-023-45940-4 (PMC10628198; doi:10.1038/s41598-023-45940-4)
Supplement: Supplementary file 1 — Supplementary Information. [file 41598_2023_45940_MOESM1_ESM.pdf]

Supplementary material: Optical valley separation in two-dimensional semimetals with tilted Dirac cones

Andrew Wild,* Eros Mariani,† and M. E. Portnoi‡

Physics and Astronomy, University of Exeter, Stocker Road, Exeter EX4 4QL, United Kingdom

I. ANALYTIC EXPRESSION FOR THE DISTRIBUTION OF PHOTOEXCITED CARRIERS IN TILTED DIRAC CONES

In this section, we present the expression for the distribution of photoexcited carriers $\mathcal{F}^{\xi}(\varphi_{\mathbf{q}})$ for carriers with valley index ξ as a function of wavevector angle $\varphi_{\mathbf{q}}$. The photoexcited electrons fall on the perimeter of an ellipse in wavevector space governed by Eq. (4) of the main text. We can express the Cartesian co-ordinates of this ellipse through the wavevector angle:

$$q_{x,c}(\varphi_{\mathbf{q}}) = \frac{\pi\nu\cos(\varphi_{\mathbf{q}})}{v_{\mathrm{F}}\sqrt{\eta^2\cos^2(\varphi_{\mathbf{q}})+\sin^2(\varphi_{\mathbf{q}})}}, \tag{1}$$

and

$$q_{y,c}(\varphi_{\mathbf{q}}) = \frac{\pi\nu\sin(\varphi_{\mathbf{q}})}{v_{\mathrm{F}}\sqrt{\eta^2\cos^2(\varphi_{\mathbf{q}})+\sin^2(\varphi_{\mathbf{q}})}}. \tag{2}$$

Combining Eqs.(2) and (4)-(6) of the main text and solving the resultant integral yields an expression for the distribution of photoexcited carriers as a function of the wavevector angle

$$\begin{aligned}\mathcal{F}^{\xi}(\varphi_{\mathbf{q}}) =& \frac{\alpha}{2}\frac{\eta^2\sin^2(\varphi_{\mathbf{q}}-\theta)}{\left[\eta^2\cos^2(\varphi_{\mathbf{q}})+\sin^2(\varphi_{\mathbf{q}})\right]^2}\left(1-\left\{1+\exp\left[\frac{h\nu\xi\eta\gamma\cos(\varphi_{\mathbf{q}})}{2k_{\mathrm{B}}T\sqrt{\eta^2\cos^2(\varphi_{\mathbf{q}})+\sin^2(\varphi_{\mathbf{q}})}}+\frac{h\nu}{2k_{\mathrm{B}}T}-\frac{\mu}{k_{\mathrm{B}}T}\right]\right\}^{-1}\right)\times \\ & \left\{1+\exp\left[\frac{h\nu\xi\eta\gamma\cos(\varphi_{\mathbf{q}})}{2k_{\mathrm{B}}T\sqrt{\eta^2\cos^2(\varphi_{\mathbf{q}})+\sin^2(\varphi_{\mathbf{q}})}}-\frac{h\nu}{2k_{\mathrm{B}}T}-\frac{\mu}{k_{\mathrm{B}}T}\right]\right\}^{-1}.\end{aligned} \tag{3}$$

II. ANALYTIC EXPRESSION FOR THE POLARIZATION OF VALLEY CARRIERS

In this section, we provide an analytic expression for the degree of valley polarization for photoexcited carriers that propagate to the right-hand side of the light spot ($\mathcal{S}_R$). As the Dirac cones in either valley are tilted in opposite directions, the percentage of carriers propagating to the right in valley ξ is equal to the percentage of carriers propagating to the left in valley $-\xi$ yielding the identity $\mathcal{N}_R^{\xi} = \mathcal{N}_L^{-\xi}$. Utilizing this expression, and the identity $\mathcal{N}_R^{\xi} + \mathcal{N}_L^{\xi} = 1$, Eq. (8) of the main text can be simplified to $\mathcal{S}_R = \xi(\mathcal{N}_R^{\xi} - \mathcal{N}_L^{\xi})$ which can be defined through the distribution of photoexcited carriers as

$$\mathcal{S}_R = \xi\frac{\int_0^{2\pi}\mathcal{F}^{\xi}(\varphi_{\mathbf{q}})\mathrm{sign}\left[v_{g,x}^{\xi}(\varphi_{\mathbf{q}})\right]\mathrm{d}\varphi_{\mathbf{q}}}{\int_0^{2\pi}\mathcal{F}^{\xi}(\varphi_{\mathbf{q}})\mathrm{d}\varphi_{\mathbf{q}}}, \tag{4}$$

where sign(...) is the sign function and $v_{g,x}^{\xi}(\varphi_{\mathbf{q}})$ is the $\hat{\mathbf{x}}$ component of the group velocity $\hat{\mathbf{x}}\cdot\mathbf{v}_g^{\xi}(\varphi_{\mathbf{q}})$. The $\hat{\mathbf{x}}$ component of the group velocity of photoexcited electrons in the conduction band can be obtained through the expression $v_{g,x}^{\xi}(\varphi_{\mathbf{q}}) = (1/\hbar)\partial_{q_x}E_+^{\xi}(\mathbf{q})$ yielding

$$v_{g,x}^{\xi}(\varphi_{\mathbf{q}}) = v_{\mathrm{F}}\eta\left[\xi\gamma+\frac{\eta\cos(\varphi_{\mathbf{q}})}{\sqrt{\eta^2\cos^2(\varphi_{\mathbf{q}})+\sin^2(\varphi_{\mathbf{q}})}}\right]. \tag{5}$$

* Corresponding author: A.Wild@exeter.ac.uk
† E.Mariani@exeter.ac.uk
‡ M.E.Portnoi@exeter.ac.uk

Substituting Eqs. (3) and (5) into Eq. (4) for type-I ($|\gamma| < 1$) Dirac cone materials incident upon by photons polarized along the crystallographic $\hat{\mathbf{y}}$ axis ($\theta = \pi/2$) yields the degree of valley polarization $\mathcal{S}_R = \mathrm{sign}(\gamma)\mathcal{S}$ where

$$\mathcal{S} = \begin{cases} -1 & -(1+|\gamma|)/2 \leq \frac{E_\mathrm{F}}{h\nu} < -(1+|\gamma|^2)/2, \\ \mathcal{S}_0, & -(1+|\gamma|^2)/2 \leq \frac{E_\mathrm{F}}{h\nu} < -(1-|\gamma|)/2, \\ \mathcal{S}_1, & -(1-|\gamma|)/2 \leq \frac{E_\mathrm{F}}{h\nu} < (1-|\gamma|)/2, \\ \mathcal{S}_2, & (1-|\gamma|)/2 \leq \frac{E_\mathrm{F}}{h\nu} < (1-|\gamma|^2)/2, \\ 1, & (1-|\gamma|^2)/2 \leq \frac{E_\mathrm{F}}{h\nu} < (1+|\gamma|)/2, \end{cases} \tag{6}$$

with

$$\mathcal{S}_0 = 1 - 2\left[\frac{\arccos(|\gamma|) + |\gamma|\sqrt{1-|\gamma|^2}}{\arccos(\psi) + \psi\sqrt{1-\psi^2}}\right], \tag{7}$$

followed by

$$\mathcal{S}_1 = \frac{2}{\pi}\left[\arccos(-|\gamma|) - |\gamma|\sqrt{1-|\gamma|^2}\right] - 1, \tag{8}$$

and

$$\mathcal{S}_2 = 2\left[\frac{\arccos(-|\gamma|) - |\gamma|\sqrt{1-|\gamma|^2}}{\arccos(\psi) + \psi\sqrt{1-\psi^2}}\right] - 1, \tag{9}$$

where $\psi = (1/|\gamma|)(2|E_\mathrm{F}|/h\nu - 1)$. In this expression we have assumed the low temperature limit where the Fermi-Dirac distributions are replaced with step functions and the chemical potential is exchanged with the Fermi energy $\mu \to E_\mathrm{F}$.

Whilst the above expressions correspond to the degree of valley polarization for photoexcited electrons in the conduction band, we can derive the same quantity for holes in the valence band. The only difference in the two derivations is that we must replace the group velocity of electrons in the conduction band with the group velocity of holes in the valence band $v^{\xi}_{g,x}(\varphi_\mathbf{q}) = (1/\hbar)\partial_{q_x} E^{\xi}_{-}(\mathbf{q})$. The resulting degree of valley polarization for holes is equivalent to the valley polarization for electrons after inverting the sign of the Fermi energy $E_\mathrm{F} \to -E_\mathrm{F}$.

For type-II ($|\gamma| > 1$) Dirac cones, assuming there is absorption which requires $|E_\mathrm{F}| < h\nu(1+|\gamma|)/2$, the degree of valley polarization for photoexcited electrons in the conduction band and holes in the valence band is equal to $\mathcal{S}_R = \mathrm{sign}(\gamma)$.
